# Quantifying how much host, pathogen, and other factors affect human protective adaptive immune responses

**DOI:** 10.3389/fimmu.2024.1330253

**Published:** 2024-02-12

**Authors:** Uri Sela, Joel M. Corrêa da Rosa, Vincent A. Fischetti, Joel E. Cohen

**Affiliations:** ^1^ Laboratory of Bacterial Pathogenesis and Immunology, The Rockefeller University, New York, NY, United States; ^2^ Department of Dermatology, Icahn School of Medicine at Mount Sinai, New York, NY, United States; ^3^ Laboratory of Populations, The Rockefeller University, New York, NY, United States; ^4^ Department of Statistics, Columbia University, New York, NY, United States; ^5^ Department of Statistics, University of Chicago, Chicago, IL, United States

**Keywords:** Taylor's law, adaptive immune response, bacterial pathogenesis, correlation, data analysis, infectious diseases, essential role

## Abstract

Recognizing the “essential” factors that contribute to a clinical outcome is critical for designing appropriate therapies and prioritizing limited medical resources. Demonstrating a high correlation between a factor and an outcome does not necessarily imply an essential role of the factor to the outcome. Human protective adaptive immune responses to pathogens vary among (and perhaps within) pathogenic strains, human individual hosts, and in response to other factors. Which of these has an “essential” role? We offer three statistical approaches that predict the presence of newly contributing factor(s) and then quantify the influence of host, pathogen, and the new factors on immune responses. We illustrate these approaches using previous data from the protective adaptive immune response (cellular and humoral) by human hosts to various strains of the same pathogenic bacterial species. Taylor’s law predicts the existence of other factors potentially contributing to the human protective adaptive immune response in addition to inter-individual host and intra-bacterial species inter-strain variability. A mixed linear model measures the relative contribution of the known variables, individual human hosts and bacterial strains, and estimates the summed contributions of the newly predicted but unknown factors to the combined adaptive immune response. A principal component analysis predicts the presence of sub-variables (currently not defined) within bacterial strains and individuals that may contribute to the combined immune response. These observations have statistical, biological, clinical, and therapeutic implications.

## Introduction

Humans vary widely in their susceptibility to infection (1). Many studies identify variability in human immune genetics as a major cause of this variability (2–4). Some promote the major role of inborn errors of immunity as outweighing the possible role of pathogen variability as a source for the observed variability in human susceptibility to infection (5–7). However, recent evidence suggests that variability in the pathogen (e.g., bacterial inter-strain variability, viral inter-variant variability) contributes importantly to variability in human susceptibility to infection. For example, the Delta and Omicron variants of the SARS-CoV-2 virus carry different risk for severe outcomes i.e., different rates of hospitalization and death (8, 9), a variability that is probably a result of the high mutation rate in RNA viruses (10).

Before the current pandemic, we demonstrated that different strains of the same bacterial species can induce varied antigen-specific protective adaptive immune responses (11). We showed that the contribution to the variability in the human adaptive immune response by different bacterial strains of the same species (staphylococci) is at least as high as the variability in the immune response to the same bacterial strain by different human hosts. Considering that, during infection, at least two biological systems interact, human and pathogen, and each with its own evolving genetics, it is not surprising that the variability of the pathogen contributes importantly to the variability in immune responses.

These findings raise the question about human susceptibility to infection: How much do other factors, beyond genetic variability in humans and pathogens, influence the variability in the human protective adaptive immune response to a pathogen? One approach to answering this question is to evaluate each candidate factor with bench work and directly perform the immune assays. If executed, a different and possibly more general approach might be taken before evaluating a specific candidate. Can we predict the existence of potential variables (without specifying them) that may contribute to the variability in protective adaptive immune responses? What is the relative contribution of these predicted factors in comparison to the known ones? Answering these questions may pave the way to defining which factor(s) might have “essential” roles in the variability.

In this study, we use three statistical tools that may help to predict the possible presence of other factors contributing to human protective adaptive immune responses and to evaluate their relative contribution: Taylor’s power law (TL), principal components analysis (PCA), and a mixed-effects linear model (MLM).

Taylor’s power law (TL) describes a linear relationship between the logarithm (hereafter log, always to the base 10) of the variance of the population size or the density of a species and the log of the mean of population size or density (12). While this relationship was demonstrated in several thousand publications (13), its application in human immunology is limited [Pp. 399-403 of reference 13]. Here, (i) we examine whether our previous findings that demonstrate variability in the protective adaptive immune responses (T and B cell) to the human pathogen *Staphylococcus aureus* (11) can be modeled using TL, and (ii) we analyze the result using MLM and PCA, a dimensionality reduction method, to predict that other variables may contribute to variability in the human protective adaptive immune response to a pathogen.

## Results

Peripheral blood mononuclear cells from 10 healthy human donors were exposed to 16 different strains of *S. aureus* (11). For each donor-strain pair, we measured four adaptive immune responses: i) T cell proliferation, ii) interferon gamma (IFNγ) expression by proliferating T cells, iii) B cell proliferation, and iv) immunoglobulin G (IgG) expression by proliferating B cells.

For each response separately, for each strain separately, we calculated the mean and the variance of the response across the 10 donors and plotted the log variance (y axis) against the log mean (x axis) of the resulting 16 (mean, variance) pairs. Also, for each response separately, and for each donor separately, we calculated the mean and the variance of the response across the 16 strains and plotted the log variance (y axis) against the log mean (x axis) of the resulting 10 (mean, variance) pairs.

**Figure 1:**
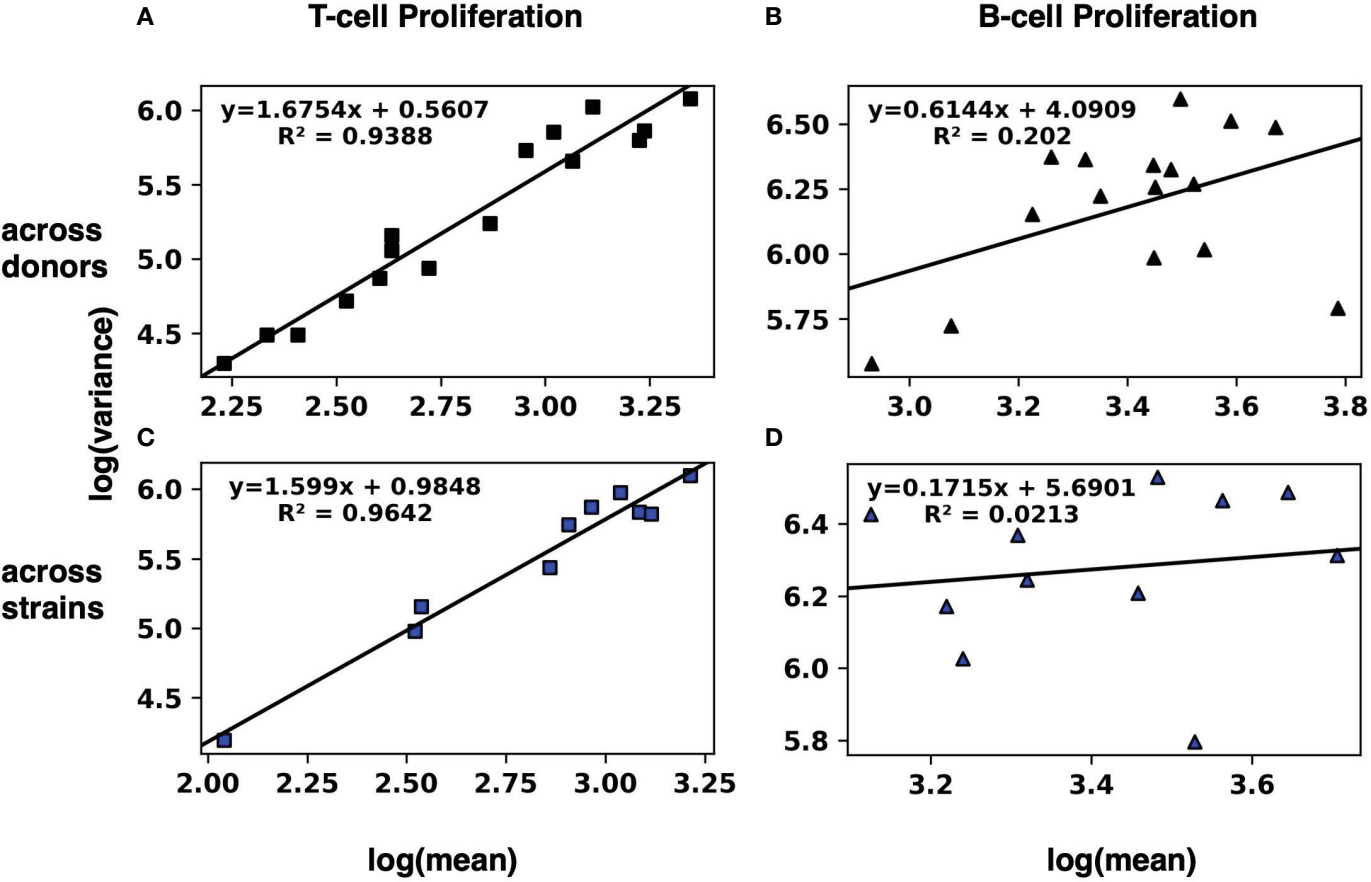
Modeling the human adaptive immune response to *S. aureus* with Taylor’s law. The mean and the variance of T-cell **(A, C)** and B-cell **(B, D)** proliferation across 10 donors **(A, B)** and across 16 strains **(C, D)** were calculated. Then the log variance (y axis) was plotted against the log mean (x axis) of the resulting 16 (log mean, log variance) pairs across donors, and a straight line was fitted by least squares. Similarly, the 10 pairs (log mean, log variance) across strains were plotted, and a straight line was fitted by least squares. Results for IFNγ and IgG expression by T-cells and B-cells, respectively, are in **Supplementary Figure 1**.

To evaluate whether these results can be modeled with TL, we fitted by least squares a straight line for T cell proliferation and IFNγ expression for the values across the 10 donors (**Figure 1A**; **Supplementary Figure 1A**, respectively) as well as across the 16 strains (**Figure 1C**; **Supplementary Figure 1B**, respectively). Similarly, we fitted a straight line for B cell proliferation and IgG expression across the 10 donors (**Figure 1B**; **Supplementary Figure 1C**, respectively), as well as across the 16 strains (**Figure 1D**; **Supplementary Figure 1D**, respectively).

The linear regressions for T cell proliferation and IFNγ expression both across donors and across strains had squared correlation coefficients R^2^ greater than 0.85, suggesting a strong linear relationship of log variance to log mean. Visual inspection of the plots is consistent with a linear relationship. By contrast, the linear regressions for B cell proliferation and IgG expression (both across donors and across strains) had much lower squared correlation coefficients R^2^ (**Table 1**).

**Table 1:** R^2^ values for log variance as a linear function of log mean, across donors and across strains, for four human immune responses.

	T-cell	IFNγ	B-cell	IgG
Across donors	0.9388*	0.8598*	0.2020	0.4270
Across strains	0.9642*	0.9567*	0.0213	0.0930

*R^2^ > 0.85.

**Figure 2:**
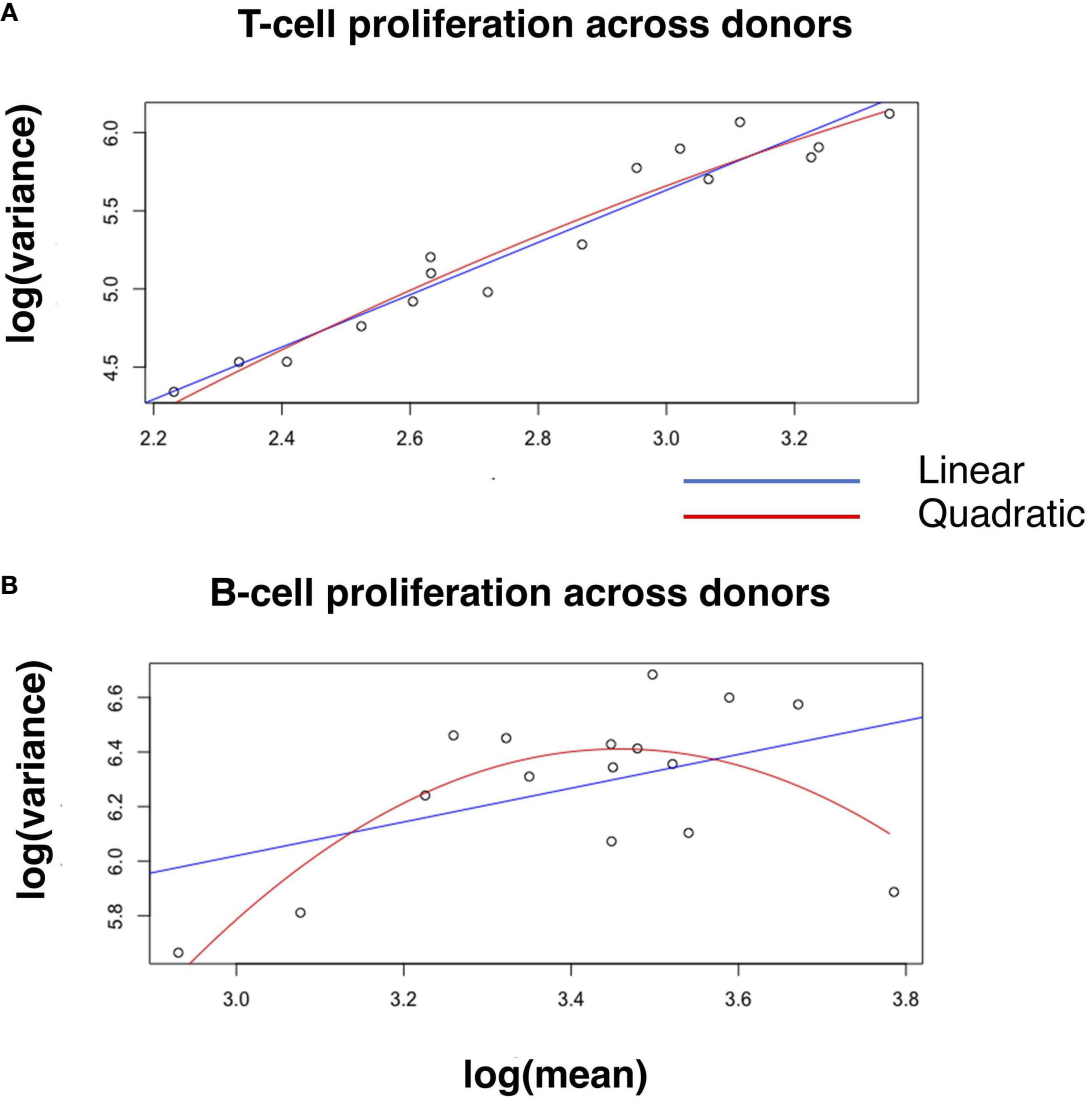
Linear and quadratic fit of the relationship between log-variance and log-mean in cell proliferation. Blue and red lines in the scatter plot represent the linear and quadratic fit, respectively, for T-cell proliferation **(A)** and B-cell proliferation **(B)** across donors. See **Table 2** for statistical differences between the two fits, and **Supplementary Figure 2** for other variables across donors and strains.

To test quantitatively whether the linear relationship of log variance to log mean is better than plausible alternatives, we evaluated whether an additional quadratic term significantly better captures the relationship between log mean and log variance (Materials and Methods, Equation 1). Adding a quadratic term does not significantly improve the coefficient of determination (R^2^) across donors and across strains for T cell proliferation (**Figure 2A** and **Supplementary Figure 2**), IFNγ expression and IgG expression (**Table 2**; **Supplementary Table 2**; **Supplementary Figure 2**). However, for B cell proliferation across donors (**Figure 2B**), a quadratic fit is better than the linear one (B_2_ is statistically significantly negative with a p-value=0.005). Thus, at least for T cell proliferation and IFNγ expression, both across donors and across strains, a linear fit is the best fit among the models we considered.

**Table 2:** Linear and quadratic fits for T-cell and B-cell proliferation across donors.

T-Cells across donors (Linear)					
Coefficients	Estimate	Std.Error	t-value	p-value	R^2^
**B0**	0.609	0.323	1.88	0.081	0.939
**B1**	1.675	0.114	14.66	0.000	
T-Cells across donors (Quadratic)					
Coefficients	Estimate	Std.Error	t-value	p-value	R^2^
**B0**	-2.315	2.956	-0.783	0.447	0.943
**B1**	3.795	2.134	1.779	0.099	
**B2**	-0.379	0.381	-0.995	0.338	
B-Cells across donors (Linear)					
Coefficients	Estimate	Std.Error	t-value	p-value	R^2^
**B0**	4.160	1.109	3.751	0.002	0.207
**B1**	0.620	0.324	1.910	0.077	
B-Cells across donors (Quadratic)					
Coefficients	Estimate	Std.Error	t-value	p-value	R^2^
**B0**	-29.284	9.910	-2.955	0.011	0.579
**B1**	20.646	5.918	3.489	0.004	
**B2**	-2.985	0.881	-3.387	0.005	

Least-squares estimates for intercept (B0), slope (B1) and curvature (B2), followed by standard error, Student's t statistic, p-value and coefficient of determination (R^2^).

To compare whether strains or donors contribute more to the variability in the immune responses, we test whether there is a significant difference between the slope across donors and the slope across strains in those cases where we verify a linear fit across donors and across strains. Because our data across donors and strains are taken from the same samples, the usual assumption that observations are independent does not hold and we cannot use the standard analysis of covariance to compare the slopes across strains and across donors from **Figure 1** and **Supplementary Figure 1**. Instead, we bootstrapped the data, repeatedly sampling with replacement from the original data to create an ensemble of statistically equivalent data sets.

**Figure 3:**
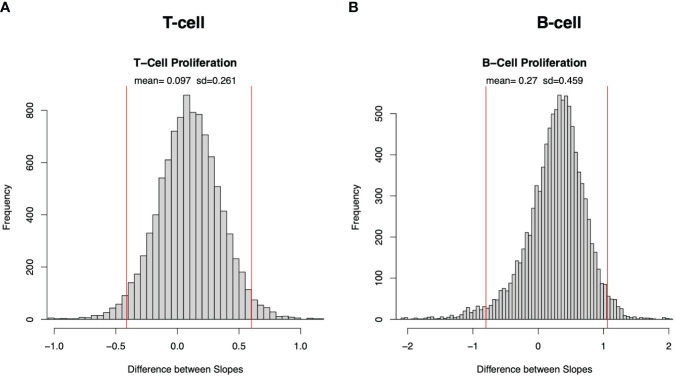
Bootstrap sampling distribution of difference in slope of Taylor’s law across donors and across strains for cell proliferation. Histogram of frequency distribution of differences between the estimated slopes across donors and across strains obtained from 5000 bootstrapped samples for T-cell **(A)** and B-cell **(B)** proliferation. Mean and standard deviation (sd) are depicted at top, and red vertical lines represent 95% confidence interval (2.5 and 97.5 percentiles are represented by left and right red lines, respectively).

**Supplementary Figure 3A** plots the histograms of the slopes resulting from bootstrapping of T cell proliferation across donors (red) and strains (blue). **Figure 3A** plots the histogram of the slope across donors minus the slope across strains and demonstrates that there is no significant difference in slopes for T cell proliferation. This lack of significant difference in slopes of TL means that there is no significant difference in the fractional increase of the variance associated with a fractional increase of the mean T cell proliferation whether the means and variances are calculated across donors or across strains. Similarly, **Figure 3B** demonstrates no significant difference in slopes of B cell proliferation across donors and strains and **Supplementary Figure 3** demonstrates similar findings for IFNγ (**Supplementary Figure 3D**) and IgG expression (**Supplementary Figure 3H**). Thus, in all four immune responses, this analysis does not detect a significant difference in the rate of increase of the variability in the adaptive immune response resulting from given increases in the mean responses associated with donors or strains. However, this finding of no difference in the rate of increase of variability is a different question from whether, at a given fixed average of the immune response level, donors or strains contribute more to the variability of the immune response. We address that question below.

The similarity between the slopes across donors and the slopes across strains is not a tautologous or automatic consequence of using the same array of data to estimate both versions of TL. To prove this, the **Supplementary Information Text** and **Supplementary Figures 4**, **5** give two artificial examples of numerical arrays with 16 rows (analogous to strains) and 10 columns (analogous to donors) in which the slope of TL across rows is large and positive while the slope of TL down columns is quite negative.

Where the data support a linear relationship (**Figures 1**, **2** and **Table 1**) between log variance and log mean, we now ask whether the magnitudes of the slopes are more consistent with a Poisson (purely random) distribution (in which the mean and variance are the same, so the slope equals 1), or are more consistent with overdispersion (in which variance is greater than mean, with a slope greater than 1) which suggests some additional (yet unobserved) source of variation beyond purely Poisson variation (14). As shown in **Table 3**, the slope is greater than 1 for T cell proliferation across donors and across strains and IFNγ expression across strains. This result strongly suggests that in addition to the variation induced by donors and strains, other variable(s) may contribute to the variability in T cell proliferation and IFNγ expression.

**Table 3:** Slope plus or minus 95% confidence interval, across donors and across strains, for four human immune responses.

	T-cell Prolif	IFNγ	B-cell Prolif	IgG
Across donors	1.6754 ± 0.2452*	1.8608±0.4308	0.6144±0.700	0.5588±0.3710
Across strains	1.599±0.2511*	1.6805±0.2914*	0.1715±0.8762	0.4159±1.0591

^*^1 < Slope ±95% CI < 2.

Except for IFNγ across donors, the slope of log variance as a function of log mean is also less than 2. When that slope is less than 2, the coefficient of variation (standard deviation divided by the mean) decreases as the mean increases, suggesting that the variation in the immune response is increasingly controlled or regulated as the average strength of the immune response increases.

**Figure 4:**
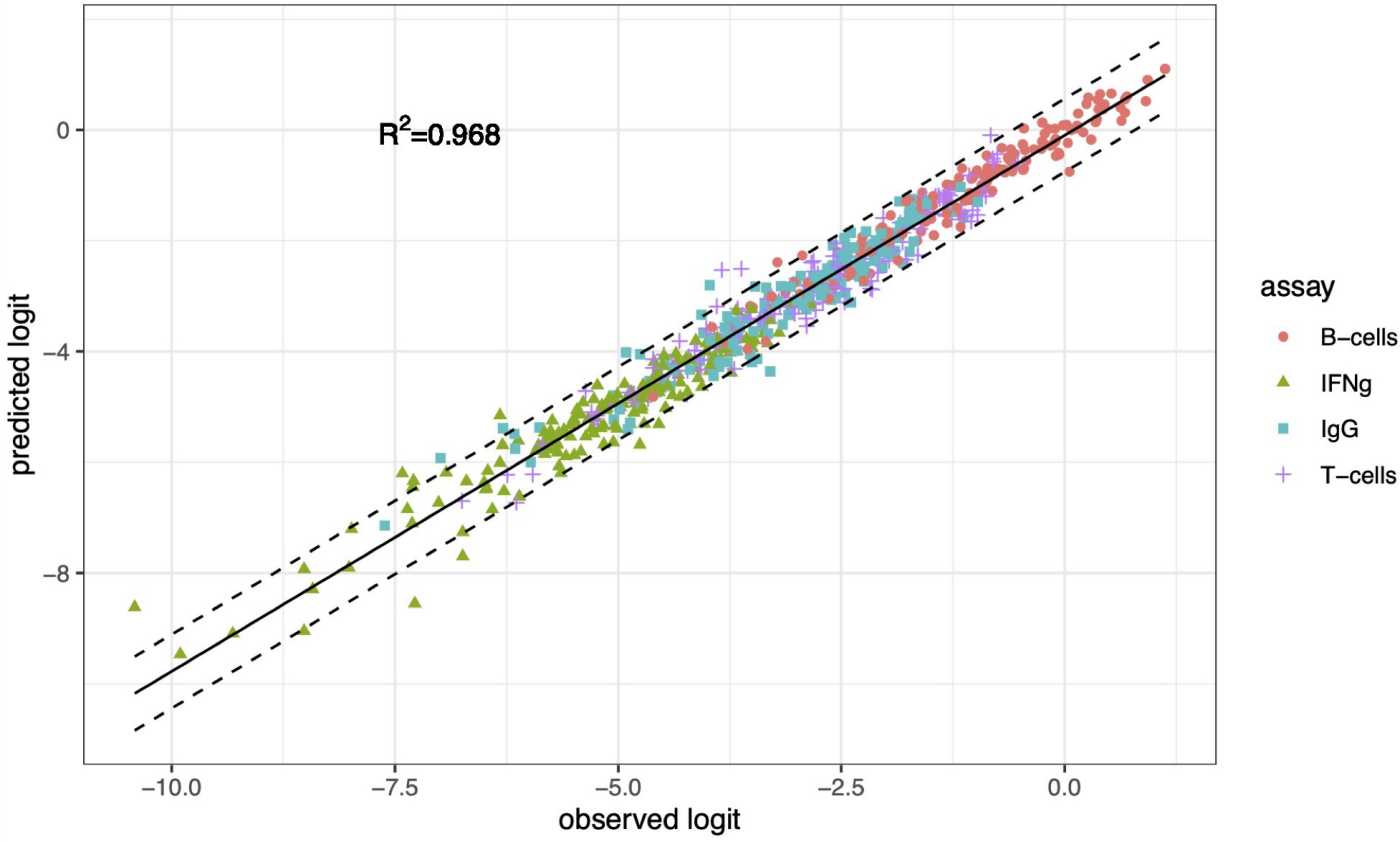
The association between logit-transformed data (observed logit) and predictions (predicted logit) from a mixed-effects linear model. The MLM includes assay, strain, donor, and two-way interactions of strain with assay and of donor with assay as fixed effects and random effects for bacteria and donor, and interactions donor with assay and bacteria with assay. The dashed lines are 95% prediction intervals. R^2^ is the coefficient of determination.

TL may predict the presence of other contributing factors, as it does here, but it does not quantify their relative contribution. Finding slopes greater than 1 prompted us to compare the relative contribution to the combined adaptive immune response of bacterial variability between strains within the *S. aureus* species with the contribution of the inter-individual-host variability. To this end, we fitted a MLM in which the continuous dependent variable is the logit-transformed percentage of 10,000 cells that responded, and the independent categorical variables are the four assays (T-cell and B-cell proliferation, IFNγ and IgG expression), the 16 bacterial strains, the 10 donors, the pairwise interactions of strains with assays, and the pairwise interactions of donors with assays (Materials and Methods). The predicted dependent values from the MLM correlated very strongly with the logit-transformed response percentages in the data (coefficient of determination R^2^ = 0.968, **Figure 4**). The components of variance from this MLM (**Table 4**) demonstrate that beyond the relatively large contribution of intra-species inter-strain variability (31.1%) and inter-individual variability (54.9%), other currently not known potential “Residual” sources contribute 15% to the combined adaptive immune response.

**Table 4:** Variance components extracted from the mixed-effects model.

Source of Variation	Variance (Random Effect)	Std. Dev.	% Component of Variance
Bacteria*	0.718	0.847	31.1
Donors^	1.31	1.15	54.9
Residual	0.36	0.6	15

*Total Bacterial contribution in isolation plus their interaction with assay.

^Total Donors contribution in isolation plus their interaction with assay.

The relatively high contribution of interindividual host variability and inter-strain variability to the combined adaptive immune response (**Table 4**) raises the question whether potential additional sources (or “sub-variables”) contribute to the variance within the groups of donors and or bacteria. To explore this possibility, we performed PCA (**Table 5**). The first two components in combination explain most of the variance in each group (across all four assays, at least 84% of the variation among the donors and at least 79% of the variation among the bacteria). Further investigation may reveal specific features that play a major role in explaining the principal components.

**Table 5:** Percentage of variance explained by components 1, or 2, or 1 and 2 from principal components analysis.

Variable	Observation	Component	B cell	T cell	IFNγ	IgG
**Donor**	**Bacteria**	**1**	71.4183	86.4368	64.1242	75.3673
**Donor**	**Bacteria**	**2**	12.8487	5.4403	19.8557	10.2623
**Donor**	**Bacteria**	**1 and 2**	**84.267**	**91.8772**	**83.9799**	**85.6296**
**Bacteria**	**Donor**	**1**	79.2881	82.3364	55.9695	74.5172
**Bacteria**	**Donor**	**2**	8.3626	6.1720	22.6551	11.8781
**Bacteria**	**Donor**	**1 and 2**	**87.6507**	**88.5085**	**78.6246**	**86.3952**

Boldface lines 3 and 6 combine and summarize lines 1, 2 and 4, 5 respectively.

## Discussion

Even if a candidate factor A correlates strongly with protective response B, to demonstrate that factor A has an “essential role” in human susceptibility to infection, two additional steps are required: (i) to evaluate whether additional factors may contribute to response B; and (ii) to evaluate the relative contribution of factor A to response B. While factor A may contribute to outcome B, its contribution relative to other factors may be either negligible or essential. Therefore, to this end, in this paper, we use an approach that first predicts the presence of potential other factors that may contribute to the protective adaptive immune response beyond the known factors (human and bacterial). Then, we analyze statistically the relative contributions of different factors (including newly predicted ones) to human protective adaptive immune responses (**Table 6**).

**Table 6:** Statistical tools useful to determine whether a proposed factor has an “essential role” in an immune response.

Statistical test	Conclusions
Taylor’s law (TL)	Points to the existence of other variables
Logit transformation/mixed effects model (MLM)	Points to the existence of other variables (residuals) and their relative contribution compared to the known variables
Principal components analysis (PCA)	Points to the existence of additional sub-variables within the known variables

TL and a MLM can be used to suggest the potential presence of additional variables, beyond the known factors, that may contribute to the human immune response. The MLM can estimate their relative contribution (both the known factors and the predicted additional ones). PCA shows that the variability within each known factor, here donors and bacteria, may be decomposed into finer sub-variables. For example, the relatively high contribution of the interindividual host variability (54.9%, **Table 4**) to the combined adaptive immune response may indicate that host variability, especially human immune genetics, has an essential contribution. PCA raises the possibility that the host contribution results in part, at least, from several potential sub-variables, such as: interindividual variability in Vitamin D level, a vitamin that is essential for proper immune response during infection (15); variability in B12 and folic acid, zinc and other trace elements that are necessary for proper immune system function and for eliminating the infection (16–18); variability in the microbiome; variability in body mass index [obesity impairs immune function, leukocyte count and adaptive immune response (19–21)]; and perhaps others.

A similar analysis should be applied to other potential sub-variables that might be included in inter-strain variability. As all bacterial strains were grown under the same nutritional conditions and temperature, our current knowledge suggests that most effects contributed by inter-strain variability probably originate from genetic variation among strains.

By using TL, we have shown a previously unrecognized, systematic pattern in the variability of some human immune responses. Specifically, for T cell proliferation and IFNγ expression, the variance of the fraction of responsive human blood cells increases as a power of the corresponding mean responses, whether mean and variance are calculated across human subjects or across bacterial strains within *S. aureus*. A practical statistical implication of this finding is that ordinary analysis of variance (ANOVA) cannot be used to compare the responses of two groups of such observations because ANOVA assumes equal variances in all groups being compared. TL shows how the variance can be rescaled to render ANOVA applicable if the mean response varies among groups. Biologically, TL shows that the higher the immune response, the more variable it is, in an orderly way. Consequently, there is more room for evolution, as evolution depends on variation (22, 23).

The statistical approaches proposed in our study have independent variables (predictors) and dependent variables (outcomes). Our examples currently have only categorical independent variables such as various bacterial strains or different human donors, and only continuous dependent variables such as % T cell proliferation response. The statistical approaches can potentially be extended in future work to include, for example, categorical independent variables and categorical dependent variables, e.g., the relative contribution of a mutation in a human immune gene (independent) to death or survival during infection (dependent).

Our results may also have medical and therapeutic implications. The relatively high variability in protective adaptive immune responses to different pathogenic bacterial strains may lead to variability in the clinical outcome. In the future, an understanding of inter-strain variability may affect how we treat an infection. For example, in the case of *S. aureus* bacteremia, two main factors that affect the way we treat the patients are the antibiotic sensitivity of the specific strain, and whether the bacteremia is defined as complicated (in which case, 4 weeks instead of 2 weeks (24) of intravenous antibiotic treatment are warranted). However, currently, the strain-dependent variability in intensity of the protective adaptive immune response is not yet part of the decision algorithm used for treatment. Yet, if it were, a treatment tailored to the specific characteristics of the infecting strain’s genetics would result in a more predictable outcome.

## Materials and methods

### Blood and bacterial samples

For details see Sela et al. (11). Briefly, blood samples were collected from 10 healthy donors according to our IRB guidelines. PBMCs were isolated and reacted against each of 16 *Staphylococcus aureus* strains from our lab repository. T cell and B cell proliferation as well as IFNγ expression by T cells and IgG expression by B cells were measured with flow cytometry.

### Quadratic variance function

For each response, and for each alternative across donors or across strains, X = log mean and Y = log variance. The quadratic model is [25; see page 388, Equation (14)]:


(1) $$\text{Y}={\text{B}}_{0}+{\text{B}}_{1}*\text{X}+{\text{B}}_{2}*{\text{X}}^{2}+\text{Error}$$

### Bootstrapping

Bootstrap was performed to assess differences between slopes estimated across donors and bacteria strains. 5000 resamples were drawn using the function “sample” in R software, setting a seed parameter for reproducibility. In each resample, we fitted two linear regressions modeling the log variance as a function of the log mean of responses; one across donors, the other across strains. The 95% confidence limits were derived from computing the 2.5 and 97.5 percentiles of differences between slopes generated in the resamples.

### Mixed effect models and components of variance

We fitted a linear mixed-effects model to logit-transformed responses (percentage of donor blood cells responding). The model was fitted by the Restricted Maximum Likelihood method implemented in the “lmer” R package. In R notation, our model was:


lmer(logitp ∼ 1+bacteria+assay+donors+bacteria:assay+donors:assay+(1|bacteria)+(1|donors)+(1|bacteria:assay)+(1|donors:assay).

The fixed effects were bacteria, assay, donors, bacteria:assay interaction, and donors:assay interaction. The random effects were (1|bacteria) + (1|donors) + (1|bacteria:assay) + (1|donors:assay). Using the intra-class correlation coefficient, we attributed to donors the components of variance due to both terms (1|donors) + (1|donors:assay). Similarly, the components of variance we attributed to bacterial strains were due to both terms (1|bacteria) + (1|bacteria:assay) (**Table 4**).

We fitted a simple linear regression to evaluate how close the predicted values from the mixed model were to the observed logit-transformed responses. We also computed the coefficient of determination R^2^ and the 95% prediction interval (**Figure 4**) with the formulation described in [26, pp. 226-227].

## Data availability statement

The original contributions presented in the study are included in the article/**Supplementary Material**. Further inquiries can be directed to the corresponding authors.

## Ethics statement

The studies involving humans were approved by RU IRB. The studies were conducted in accordance with the local legislation and institutional requirements. The participants provided their written informed consent to participate in this study.

## Author contributions

US: Conceptualization, Data curation, Formal analysis, Investigation, Methodology, Project administration, Writing – original draft, Writing – review & editing, Resources, Supervision, Validation, Visualization. JCR: Data curation, Formal analysis, Software, Investigation, Methodology, Visualization, Writing – review & editing. VAF: Funding acquisition, Writing – review & editing. JEC: Conceptualization, Formal analysis, Investigation, Methodology, Supervision, Validation, Visualization, Writing – review & editing.

## Supplementary material

Supplementary Figure 1 Modeling the adaptive immune response to *S. aureus* with Taylor’s law for IFNγ **(A, B)** and IgG expression **(C, D)** across donors **(A, C)** and strains **(C, D)**.

Supplementary Figure 2 Linear and quadratic fit of the relationship between log-variance and log-mean in cell proliferation and expression. Blue and red lines represent the linear and quadratic fit, respectively, for T-cell **(A)** and B-cell **(B)** proliferation across strains, IFNγ **(C, D)** and IgG **(E, F)** expression across donors **(C, E)** and strains **(D, F)**.

Supplementary Figure 3 Bootstrap sampling distribution for slopes and difference between slopes across donors and across strains. Histogram of frequency distribution of 5000 bootstrapped slopes across donors (left panel in **A, C, E, G**) and across strains (right panel in **A, C, E, G**) in T-cell **(A)**, B-cell **(E)** proliferation, IFNγ **(C)**, and IgG **(G)** expression. Histogram of frequency distribution of 5000 bootstrapped differences **(B, D, F, H)** between estimated slopes across donors and across strains in T-cell **(B)** B-cell **(F)** proliferation, IFNγ **(D)** and IgG **(H)** expression. Red vertical bars delimiting 95% confidence interval (2.5 percentile and 97.5 percentile are represented by left and right red lines, respectively).

Supplementary Figure 4 First of two artificial examples of numerical arrays with 16 rows (analogous to strains) and 10 columns (analogous to donors) in which the slope of TL across rows is large and positive while the slope of TL down columns is negative.

Supplementary Figure 5 Second of two artificial examples of numerical arrays with 16 rows (analogous to strains) and 10 columns (analogous to donors) in which the slope of TL across rows is large and positive while the slope of TL down columns is negative.

Supplementary Table 2 Linear and quadratic fits of log variance as a function of log mean for T-cell and B-cell proliferation across strains and IFNγ and IgG across donors and across strains.
