# Supplementary figures and images for: Quantifying how much host, pathogen, and other factors affect human protective adaptive immune responses
Source: Front Immunol. 2024 Feb 12;15:1330253. doi: 10.3389/fimmu.2024.1330253 (PMC10895049; doi:10.3389/fimmu.2024.1330253)

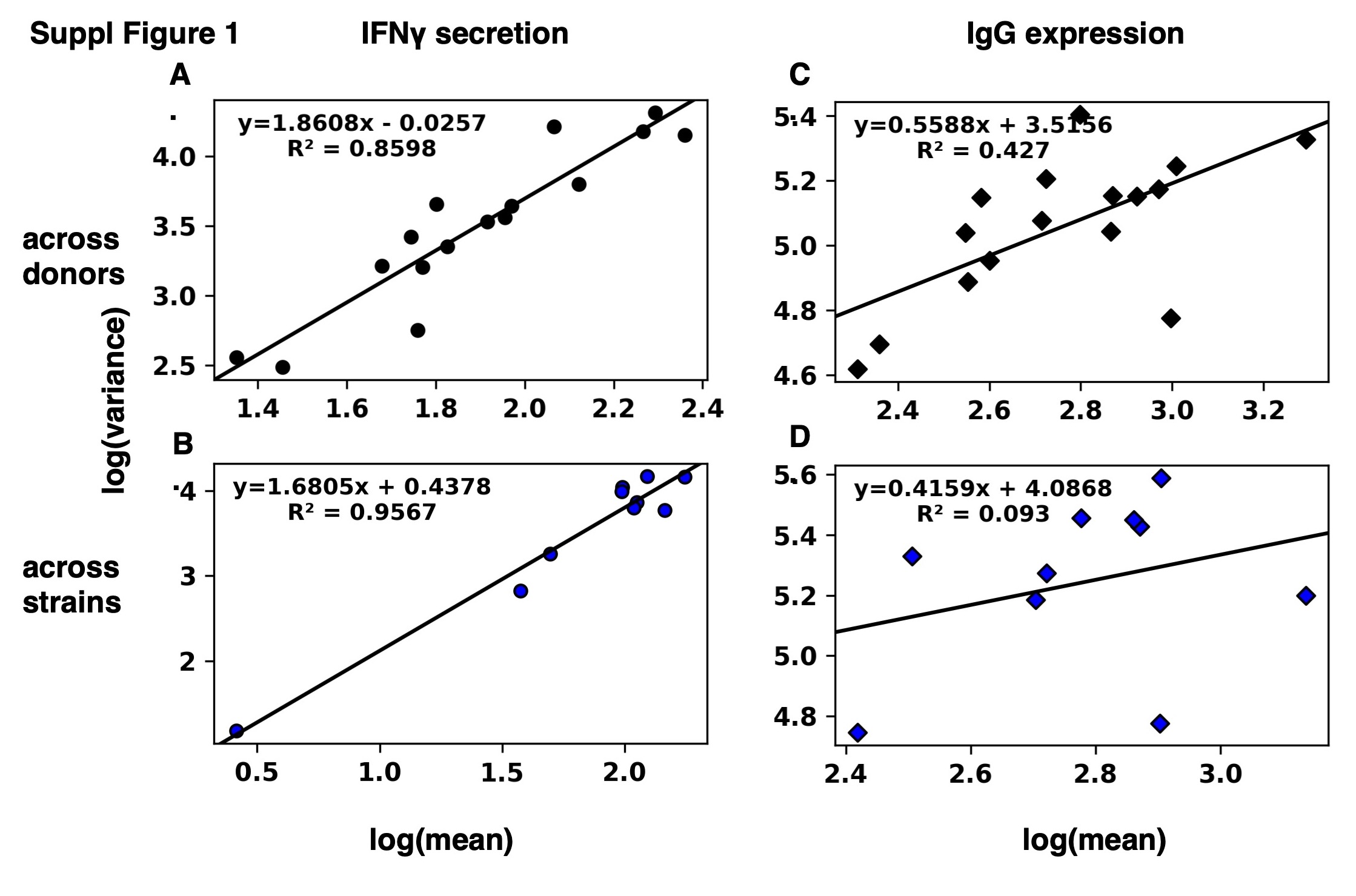

Supplement: Supplementary Figure 1 — Modeling the adaptive immune response to S. aureus with Taylor’s law for IFNγ (A, B) and IgG expression (C, D) across donors (A, C) and strains (C, D). [file Image_1.jpeg]

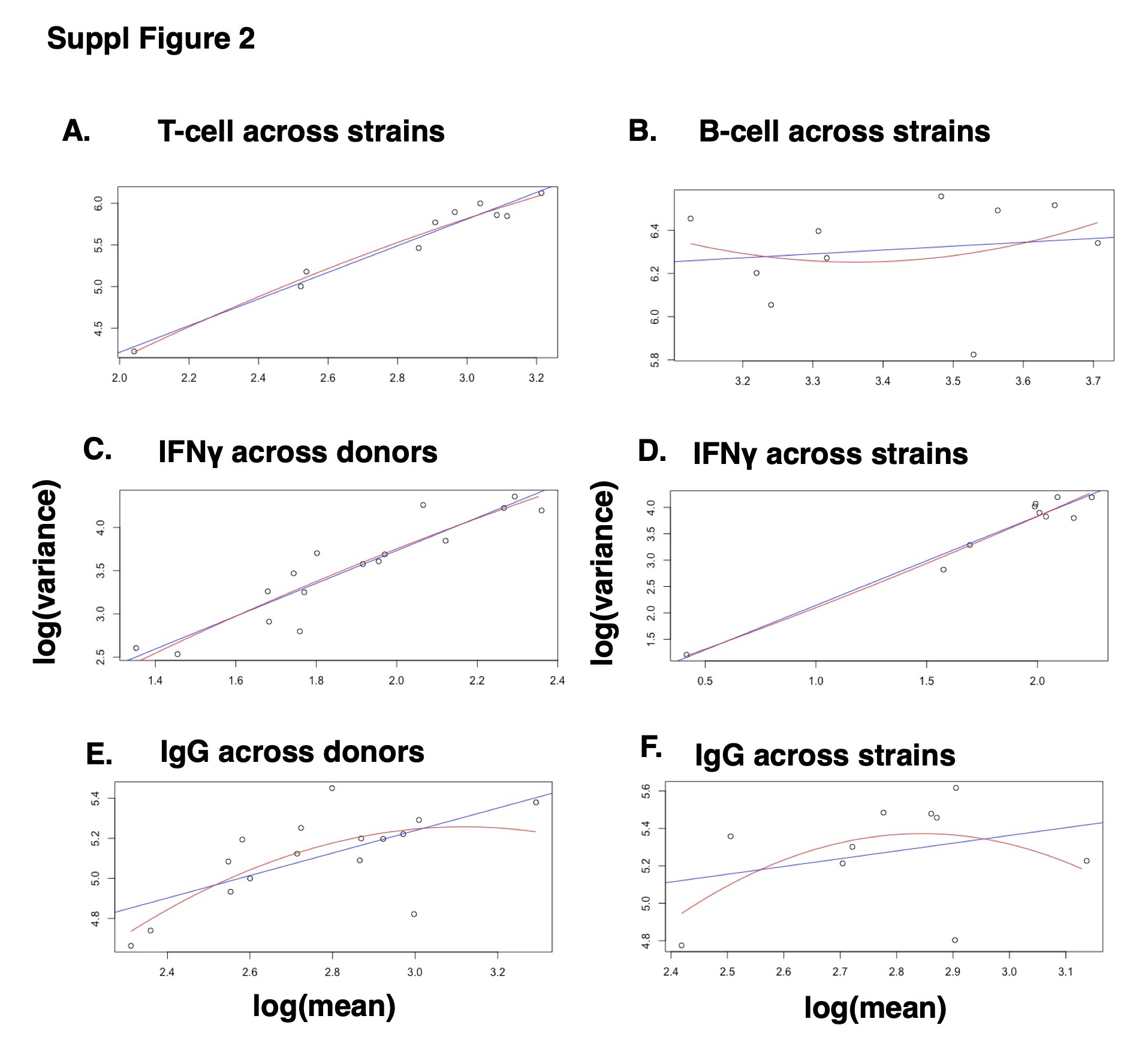

Supplement: Supplementary Figure 2 — Linear and quadratic fit of the relationship between log-variance and log-mean in cell proliferation and expression. Blue and red lines represent the linear and quadratic fit, respectively, for T-cell (A) and B-cell (B) proliferation across strains, IFNγ (C, D) and IgG (E, F) expression across donors (C, E) and strains (D, F). [file Image_2.jpeg]

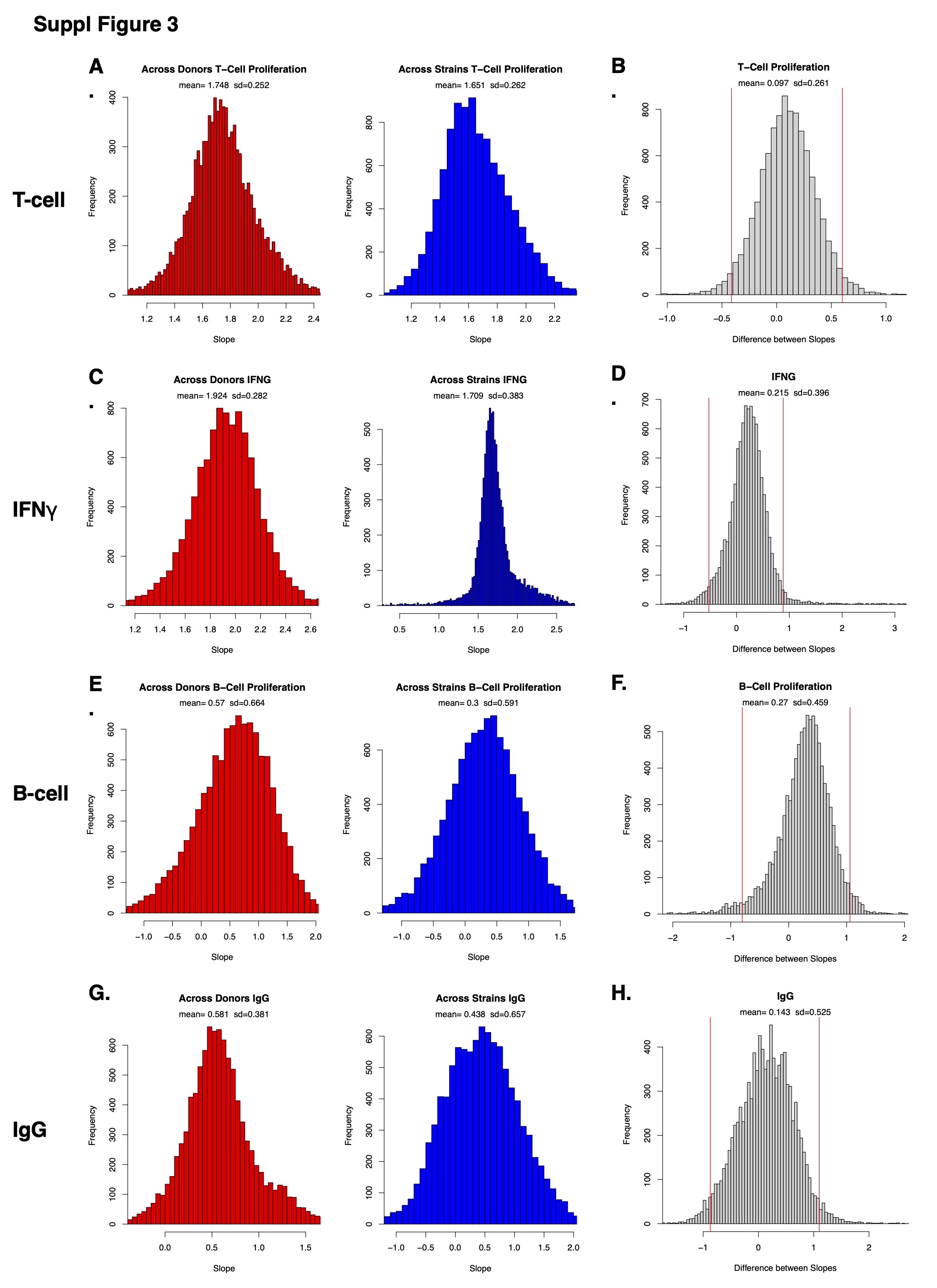

Supplement: Supplementary Figure 3 — Bootstrap sampling distribution for slopes and difference between slopes across donors and across strains. Histogram of frequency distribution of 5000 bootstrapped slopes across donors (left panel in A, C, E, G) and across strains (right panel in A, C, E, G) in T-cell (A), B-cell (E) proliferation, IFNγ (C), and IgG (G) expression. Histogram of frequency distribution of 5000 bootstrapped differences (B, D, F, H) between estimated slopes across donors and across strains in T-cell (B) B-cell (F) proliferation, IFNγ (D) and IgG (H) expression. Red vertical bars delimiting 95% confidence interval (2.5 percentile and 97.5 percentile are represented by left and right red lines, respectively). [file Image_3.jpeg]

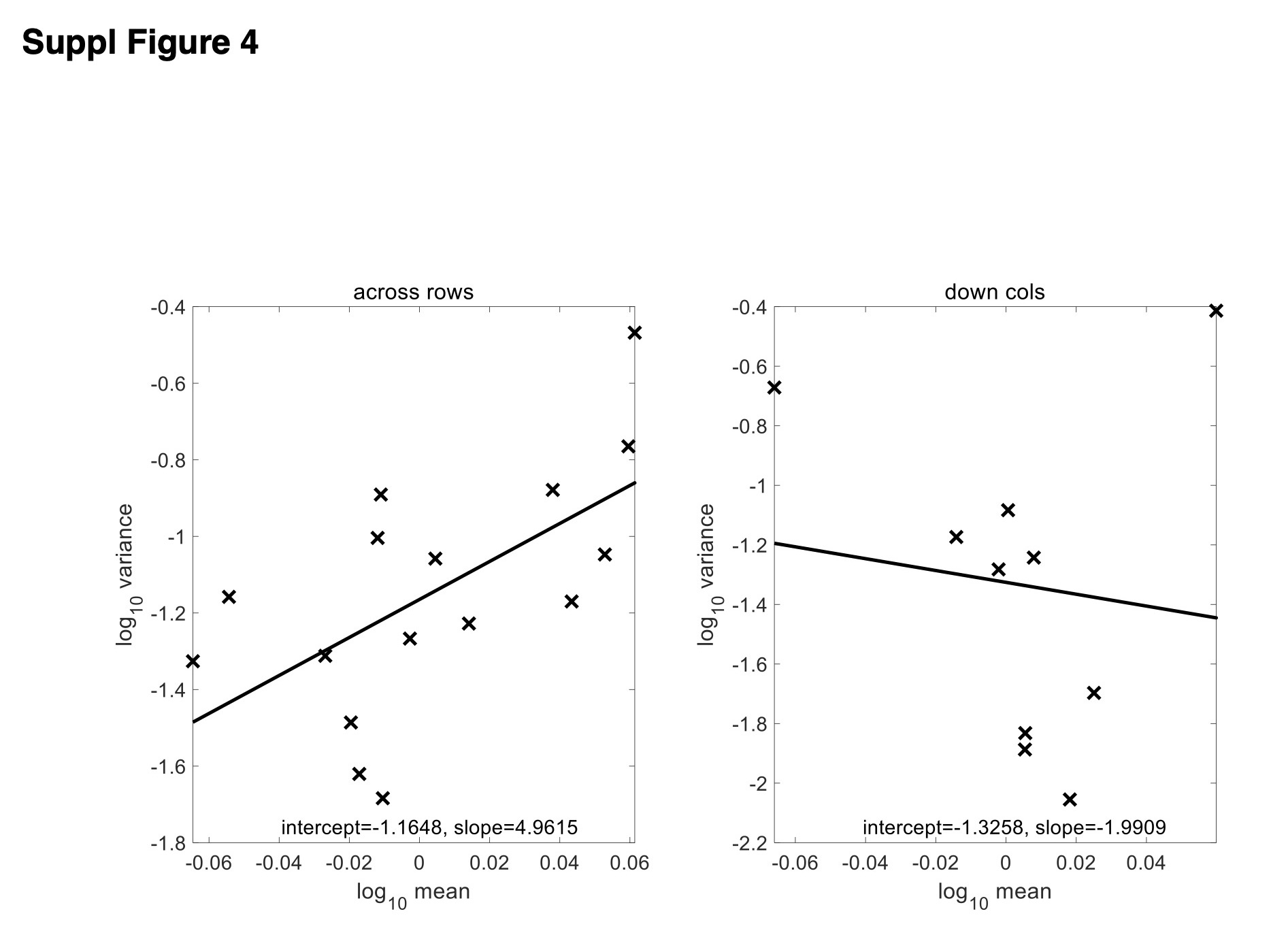

Supplement: Supplementary Figure 4 — First of two artificial examples of numerical arrays with 16 rows (analogous to strains) and 10 columns (analogous to donors) in which the slope of TL across rows is large and positive while the slope of TL down columns is negative. [file Image_4.jpeg]

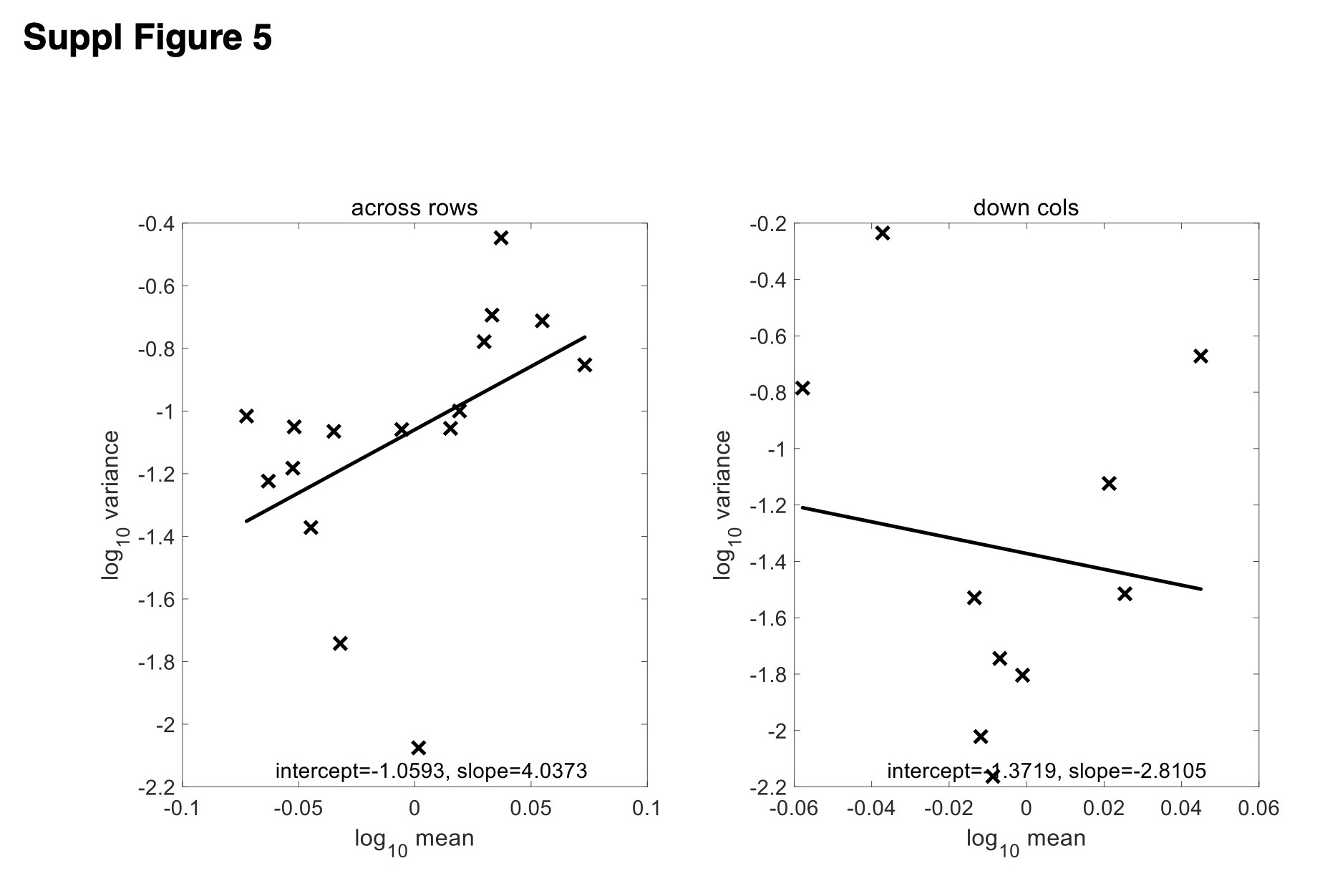

Supplement: Supplementary Figure 5 — Second of two artificial examples of numerical arrays with 16 rows (analogous to strains) and 10 columns (analogous to donors) in which the slope of TL across rows is large and positive while the slope of TL down columns is negative. [file Image_5.jpeg]
