# Supplementary material for: Quantifying how much host, pathogen, and other factors affect human protective adaptive immune responses
Source: Front Immunol. 2024 Feb 12;15:1330253. doi: 10.3389/fimmu.2024.1330253 (PMC10895049; doi:10.3389/fimmu.2024.1330253)
Supplement: Supplementary Table 2 — Linear and quadratic fits of log variance as a function of log mean for T-cell and B-cell proliferation across strains and IFNγ and IgG across donors and across strains. [file Table_2.docx]

**Suppl Table 2. Linear and quadratic fits of log variance as a function of log mean for T-cell and B-cell proliferation across strains and IFNγ and IgG across donors and across strains.**

| **T-Cells across strains (Linear)** | | | | | |
| --- | --- | --- | --- | --- | --- |
| **Coefficients** | Estimate | Std.Error | t-value | p-value | R^2^ |
| **B0** | 1.013 | 0.311 | 3.26 | 0.012 | 0.964 |
| **B1** | 1.599 | 0.109 | 14.66 | 0.000 |  |
| **T-Cells across strains (Quadratic)** | | | | | |
| **Coefficients** | Estimate | Std.Error | t-value | p-value | R^2^ |
| **B0** | -1.077 | 2.185 | -0.493 | 0.637 | 0.968 |
| **B1** | 3.215 | 1.676 | 1.918 | 0.097 |  |
| **B2** | -0.306 | 0.316 | -0.966 | 0.366 |  |
| **B-Cells across strains (Linear)** | | | | | |
| **Coefficients** | Estimate | Std.Error | t-value | p-value | R^2^ |
| **B0** | 5.700 | 1.399 | 4.073 | 0.004 | 0.024 |
| **B1** | 0.180 | 0.409 | 0.439 | 0.672 |  |
| **B-Cells across strains (Quadratic)** | | | | | |
| **Coefficients** | Estimate | Std.Error | t-value | p-value | R^2^ |
| **B0** | 23.611 | 33.136 | 0.713 | 0.499 | 0.063 |
| **B1** | -10.329 | 19.424 | -0.532 | 0.611 |  |
| **B2** | 1.537 | 2.840 | 0.541 | 0.605 |  |
| **IgG across donors (Linear)** | | | | | |
| **Coefficients** | Estimate | Std.Error | t-value | p-value | R^2^ |
| **B0** | 3.554 | 0.479 | 7.422 | 0.000 | 0.430 |
| **B1** | 0.562 | 0.173 | 3.248 | 0.006 |  |
| **IgG across donors (Quadratic)** | | | | | |
| **Coefficients** | Estimate | Std.Error | t-value | p-value | R^2^ |
| **B0** | -2.559 | 4.047 | -0.632 | 0.538 | 0.516 |
| **B1** | 5.015 | 2.934 | 1.709 | 0.111 |  |
| **B2** | -0.804 | 0.529 | -1.520 | 0.152 |  |
| **IFNγ across donors (Linear)** | | | | | |
| **Coefficients** | Estimate | Std.Error | t-value | p-value | R^2^ |
| **B0** | -0.067 | 0.391 | -0.170 | 0.868 | 0.860 |
| **B1** | 1.899 | 0.205 | 9.260 | 0.000 |  |
| **IFNγ across donors (Quadratic)** | | | | | |
| **Coefficients** | Estimate | Std.Error | t-value | p-value | R^2^ |
| **B0** | -1.154 | 2.339 | -0.494 | 0.630 | 0.862 |
| **B1** | 3.081 | 2.511 | 1.227 | 0.242 |  |
| **B2** | -0.314 | 0.665 | -0.472 | 0.645 |  |
| **IgG across strains (Linear)** | | | | | |
| **Coefficients** | Estimate | Std.Error | t-value | p-value | R^2^ |
| **B0** | 4.120 | 1.279 | 3.221 | 0.012 | 0.092 |
| **B1** | 0.414 | 0.459 | 0.902 | 0.393 |  |
| **IgG across strains (Quadratic)** | | | | | |
| **Coefficients** | Estimate | Std.Error | t-value | p-value | R^2^ |
| **B0** | -13.609 | 13.492 | -1.009 | 0.347 | 0.273 |
| **B1** | 13.342 | 9.808 | 1.360 | 0.216 |  |
| **B2** | -2.345 | 1.777 | -1.320 | 0.229 |  |
| **IFNγ across strains (Linear)** | | | | | |
| **Coefficients** | Estimate | Std.Error | t-value | p-value | R^2^ |
| **B0** | 0.454 | 0.246 | 1.840 | 0.103 | 0.955 |
| **B1** | 1.690 | 0.130 | 12.960 | 0.000 |  |
| **IFNγ across strains (Quadratic)** | | | | | |
| **Coefficients** | Estimate | Std.Error | t-value | p-value | R^2^ |
| **B0** | 0.579 | 0.427 | 1.354 | 0.218 | 0.955 |
| **B1** | 1.415 | 0.756 | 1.870 | 0.104 |  |
| **B2** | 0.105 | 0.284 | 0.370 | 0.722 |  |
